# Supplementary material for: Deep Learning in Thoracic Oncology: Meta-Analytical Insights into Lung Nodule Early-Detection Technologies
Source: Cancers (Basel). 2025 Feb 12;17(4):621. doi: 10.3390/cancers17040621 (PMC11853243; doi:10.3390/cancers17040621)
Supplement: Supplementary file 1 [file cancers-17-00621-s001.zip › cancers-3349900-supplementary.pdf]

Deep Learning in Thoracic Oncology: Meta-Analytical Insights into Lung Nodule Early Detection Technologies

Ting-Wei Wang, Jia-Sheng Hong, Hwa-Yen Chiu, Heng-Sheng Chao, Yuh-Min Chen, Yu-Te Wu

Table S1. PRISMA-DTA Abstract Checklist.

Section/Topic	Number	PRISMA-DTA for Abstracts Checklist Item	Reported on Page #
		TITLE and PURPOSE	
Title	1	Identify the report as a systematic review (+/- meta-analysis) of diagnostic test accuracy (DTA) studies.	1
Objectives	2	Indicate the research question, including components such as participants, index test, and target conditions.	2
		METHODS	
Eligibility criteria	3	Include study characteristics used as criteria for eligibility.	2
Information sources	4	List the key databases searched and the search dates.	2
Risk of bias & applicability	5	Indicate the methods of assessing risk of bias and applicability.	2
Synthesis of results	A1		2
		RESULTS	
Included studies	6	Indicate the number and type of included studies and the participants and relevant characteristics of the studies (including the reference standard).	2
Synthesis of results	7	Include the results for the analysis of diagnostic accuracy, preferably indicating the number of studies and participants. Describe test accuracy including variability; if meta-analysis was done, include summary results and confidence intervals.	2
		DISCUSSION	
Strengths and limitations	9	Provide a brief summary of the strengths and limitations of the evidence	2
Interpretation.	10	Provide a general interpretation of the results and the important implications.	2
		OTHER	
Funding	11	Indicate the primary source of funding for the review	NA
Registration	12	Provide the registration number and the registry name	2

Adapted From: McInnes MDF, Moher D, et al. The PRISMA-DTA Group (2018). Preferred Reporting Items for a Systematic Review and Meta-analysis of Diagnostic Test Accuracy Studies: The PRISMA-DTA Statement. JAMA. 2018 Jan 23;319(4):388-396. doi: 10.1001/jama.2017.19163.

Table S2. PRISMA-DTA Checklist.

Section/Topic	Number	PRISMA-DTA for Abstracts Checklist Item	Reported on Page #
		TITLE and PURPOSE	
Title	1	Identify the report as a systematic review (+/- meta-analysis) of diagnostic test accuracy (DTA) studies.	1
Abstract	2	Abstract: See PRISMA-DTA for abstracts.	2
		INTRODUCTION	
Rationale	3	Describe the rationale for the review in the context of what is already known.	3
Clinical role of index test	D1	State the scientific and clinical background, including the intended use and clinical role of the index test, and if applicable, the rationale for minimally acceptable test accuracy (or minimum difference in accuracy for comparative design).	3
Objectives	4	Provide an explicit statement of question(s) being addressed in terms of participants, index test(s), and target condition(s).	4
		METHODS	
Protocol and registration	5	Indicate if a review protocol exists, if and where it can be accessed (e.g., Web address), and, if available, provide registration information including registration number.	4
Eligibility criteria	6	Specify study characteristics (participants, setting, index test(s), reference standard(s), target condition(s), and study design) and report characteristics (e.g., years considered, language, publication status) used as criteria for eligibility, giving rationale.	4

Information sources	7	Describe all information sources (e.g., databases with dates of coverage, contact with study authors to identify additional studies) in the search and date last searched.	4
Search	8	Present full search strategies for all electronic databases and other sources searched, including any limits used, such that they could be repeated.	4
Study selection	9	State the process for selecting studies (i.e., screening, eligibility, included in systematic review, and, if applicable, included in the meta-analysis).	4
Data collection process	10	Describe method of data extraction from reports (e.g., piloted forms, independently, in duplicate) and any processes for obtaining and confirming data from investigators.	4-5
Definitions for data extraction	11	Provide definitions used in data extraction and classifications of target condition(s), index test(s), reference standard(s) and other characteristics (e.g. study design, clinical setting).	4-5
Risk of bias and applicability	12	Describe methods used for assessing risk of bias in individual studies and concerns regarding the applicability to the review question.	5
Diagnostic accuracy measures	13	State the principal diagnostic accuracy measure(s) reported (e.g. sensitivity, specificity) and state the unit of assessment (e.g. per-patient, per-lesion).	5
Synthesis of results	14	Describe methods of handling data, combining results of studies and describing variability between studies. This could include, but is not limited to: a) handling of multiple definitions of target condition. b) handling of multiple thresholds of test positivity, c) handling multiple index test readers, d) handling of indeterminate test results, e) grouping and comparing tests, f) handling of different reference standards	5-6
Meta-analysis	D2	Report the statistical methods used for meta-analyses, if performed.	5-6
Additional analyses	16	Describe methods of additional analyses (e.g., sensitivity or subgroup analyses, meta-regression), if done, indicating which were pre-specified.	5-6
		RESULTS	
Study selection	17	Provide numbers of studies screened, assessed for eligibility, included in the review (and included in meta-analysis, if applicable) with reasons for exclusions at each stage, ideally with a flow diagram	6
Study characteristics	18	For each included study provide citations and present key characteristics including: a) participant characteristics (presentation, prior testing), b) clinical setting, c) study design, d)target condition definition, e) index test, f) reference standard, g) sample size, h) funding sources	6-7
Risk of bias and applicability	19	Present evaluation of risk of bias and concerns regarding applicability for each study.	7
Results of individual studies	20	For each analysis in each study (e.g. unique combination of index test, reference standard, and positivity threshold) report 2x2 data (TP, FP, FN, TN) with estimates of diagnostic accuracy and confidence intervals, ideally with a forest or receiver operator characteristic (ROC) plot.	NA
Synthesis of results	21	Describe test accuracy, including variability; if meta-analysis was done, include results and confidence intervals	7-9
Additional analysis	23	Give results of additional analyses, if done (e.g., sensitivity or subgroup analyses, meta-regression; analysis of index test: failure rates, proportion of inconclusive results, adverse events).	7-9
		DISCUSSION	
Summary of evidence	24	Summarize the main findings including the strength of evidence	9-11
Limitations	25	Discuss limitations from included studies (e.g. risk of bias and concerns regarding applicability) and from the review process (e.g. incomplete retrieval of identified research).	9-11
Conclusions	26	Provide a general interpretation of the results in the context of other evidence. Discuss implications for future research and clinical practice (e.g. the intended use and clinical role of the index test)	11-12
		OTHER	
Funding	27	For the systematic review, describe the sources of funding and other support and the role of the funders	12

Adapted From: McInnes MDF, Moher D, et al. The PRISMA-DTA Group (2018). Preferred Reporting Items for a Systematic Review and Meta-analysis of Diagnostic Test Accuracy Studies: The PRISMA-DTA Statement. JAMA. 2018 Jan 23;319(4):388-396. doi: 10.1001/jama.2017.19163.

Table S3. Keywords and search results in different database

Database	Keyword	Date	Results
PubMed	(lung neoplasm OR pulmonary carcinoma OR lung cancer OR lung carcinoma) AND (screening OR detection OR diagnosis) AND (deep learning OR convolutional neural networks OR CNN) AND (computed tomography OR CT scan OR LDCT OR low dose CT OR low-dose computed tomography)	2023/11/07	848
Embase	(lung neoplasm OR pulmonary carcinoma OR lung cancer OR lung carcinoma) AND (screening OR detection OR diagnosis) AND (deep learning OR convolutional neural networks OR CNN) AND (computed tomography OR CT scan OR LDCT OR low dose CT OR low-dose computed tomography)	2023/11/07	899
Web of Science	(lung neoplasm OR pulmonary carcinoma OR lung cancer OR lung carcinoma) AND (screening OR detection OR diagnosis) AND (deep learning OR convolutional neural networks OR CNN) AND (computed tomography OR CT scan OR LDCT OR low dose CT OR low-dose computed tomography)	2023/11/07	1738

Table S4. Excluded article and reason

Title	Exclude
A lightweight neural network for lung nodule detection based on improved ghost module	Incomplete dataset specification, patient and nodule count duringn dataset splitting unclear
Construction of U-Net++ pulmonary nodule intelligent analysis model based on feature weighted aggregation	Outcome not relate to interest
Optimal lung cancer detection based on CNN optimized and improved Snake optimization algorithm	Classification task
An improved faster R-CNN algorithm for assisted detection of lung nodules	Outcome not relate to interest
A novel hybrid deep learning method for early detection of lung cancer using neural networks	Outcome not relate to interest
Controlling False-Positives in Automatic Lung Nodule Detection by Adding 3D Cuboid Attention to a Convolutional Neural Network	Outcome not relate to interest
Artificial intelligence aided diagnosis of pulmonary nodules segmentation and feature extraction	Outcome not relate to interest
Cross-domain learning for pulmonary nodule detection using Gestalt principle of similarity	Outcome not relate to interest
On using a Particle Image Velocimetry based approach for candidate nodule detection	Outcome not relate to interest
Detection of low-dose computed tomography pulmonary nodules based on 3D CNN-CapsNet	Outcome not relate to interest
Detection and classification of lung cancer computed tomography images using a novel improved deep belief network with Gabor filters	Outcome not relate to interest
Hyperparameter optimization and development of an advanced CNN-based technique for lung nodule assessment	Outcome not relate to interest
Deep learning ensemble 2D CNN approach towards the detection of lung cancer	Outcome not relate to interest
Early detection and classification of malignant lung nodules from CT images: An optimal ensemble learning	Outcome not relate to interest
A hierarchical GAN method with ensemble CNN for accurate nodule detection	Outcome not relate to interest
Detection of Lung Tumors in CT Scan Images using Convolutional Neural Networks	Outcome not relate to interest
Performance of a commercially available artificial intelligence software for the detection of confirmed pulmonary nodules and masses in canine thoracic radiography	Not CT
Influence of CT dose reduction on AI-driven malignancy estimation of incidental pulmonary nodules	Outcome not relate to interest
Machine Learning-Based Lung Cancer Detection Using Multiview Image Registration and Fusion	Not Deeplearning
Deep Fuzzy SegNet-based lung nodule segmentation and optimized deep learning for lung cancer detection	Outcome not relate to interest
Lung Cancer Classification Using Modified U-Net Based Lobe Segmentation and Nodule Detection	Outcome not relate to interest
Does the Lung Reporting and Data System Need to Be Redefined?: A Case of Missed Opportunities in Lung Cancer Screening	Case

Early detection and diagnosis of lung cancer using YOLO v7, and transfer learning	Outcome not relate to interest
ECANodule: Accurate Pulmonary Nodule Detection and Segmentation with Efficient Channel Attention	Conference
Development and evaluation of an integrated model based on a deep segmentation network and demography-added radiomics algorithm for segmentation and diagnosis of early lung adenocarcinoma	Outcome not relate to interest
Improved UNet Deep Learning Model for Automatic Detection of Lung Cancer Nodules	Outcome not relate to interest
Optimized convolutional neural network for automatic lung nodule detection with a new active contour segmentation	Outcome not relate to interest
Analysis based on machine and deep learning techniques for the accurate detection of lung nodules from CT images	Review
Auto diagnostic system for detecting solitary and juxtapleural pulmonary nodules in computed tomography images using machine learning	Outcome not relate to interest
ResDSda_U-Net: A Novel U-Net-Based Residual Network for Segmentation of Pulmonary Nodules in Lung CT Images	Outcome not relate to interest
ELCT-YOLO: An Efficient One-Stage Model for Automatic Lung Tumor Detection Based on CT Images	Outcome not relate to interest
Pulmonary Lung Nodule Detection from Computed Tomography Images Using Two-Stage Convolutional Neural Network	Outcome not relate to interest
Optimization System Based on Convolutional Neural Network and Internet of Medical Things for Early Diagnosis of Lung Cancer	Outcome not relate to interest
3-D SEH-YOLO: A YOLO-based Computer-aided Detection model for Lung Nodule Detection on Lowdose Computed Tomography	Conference
A new lung cancer detection method based on the chest CT images using Federated Learning and blockchain systems	Outcome not relate to interest
EP04.01-11 AI-based Detection on Low-Dose CT: A Focus on Augmenting Model Performance	Outcome not relate to interest
An early prediction and classification of lung nodule diagnosis on CT images based on hybrid deep learning techniques	Outcome not relate to interest
RePoint-Net detection and 3DSqU² Net segmentation for automatic identification of pulmonary nodules in computed tomography images	Outcome not relate to interest
Semi-Supervised Adversarial Learning for Improving the Diagnosis of Pulmonary Nodules	Outcome not relate to interest
Artificial Intelligence Assisted Computational Tomographic Detection of Lung Nodules for Prognostic Cancer Examination: A Large-Scale Clinical Trial	Outcome not relate to interest
Deep Learning-Based BoVW-CRNN Model for Lung Tumor Detection in Nano-Segmented CT Images	Outcome not relate to interest
Pulmonary Nodule Detection Based on Multiscale Feature Fusion	Incomplete dataset specification, patient and nodule count duringn dataset splitting unclear
A three dimensional convolutional neural network pulmonary nodule detection algorithm based on the multi-scale attention mechanism	Not English
Lung Nodule Detectability of Artificial Intelligence-assisted CT Image Reading in Lung Cancer Screening	Outcome not relate to interest
Research on classification of benign and malignant lung nodules based on three-dimensional multi-view squeeze-and-excitation convolutional neural network	Outcome not relate to interest
ISANET: Non-small cell lung cancer classification and detection based on CNN and attention mechanism	Outcome not relate to interest
Analysis of the Diagnosis Model of Peripheral Non-Small-Cell Lung Cancer under Computed Tomography Images	Retracted
An Enhanced Priori Knowledge GAN for CT Images Generation of Early Lung Nodules with Small-Size Labelled Samples	Retracted
SC-Dynamic R-CNN: A Self-Calibrated Dynamic R-CNN Model for Lung Cancer Lesion Detection	Outcome not relate to interest
A Neural Network and Optimization Based Lung Cancer Detection System in CT Images	Outcome not relate to interest
SE-GAN: A 3D conditional generative adversarial network with concurrent squeeze-and-excitation blocks for lung nodule segmentation	Outcome not relate to interest
How Many Private Data are Needed for Deep Learning in Lung Nodule Detection on CT Scans? A Retrospective Multicenter Study	Incomplete dataset specification, patient and nodule count duringn dataset splitting unclear
Segmentation and Classification of Lung Cancer byUsing Neural Network for Biomedical Images	Outcome not relate to interest
LIDP: A Lung Image Dataset with Pathological Information for Lung Cancer Screening	Dataset
Automatic lung cancer detection from CT image using improved deep neural network and ensemble classifier	Outcome not relate to interest
Computer-Aided Detection of Human Lung Nodules on Computer Tomography Images via Novel Optimized Techniques	Outcome not relate to interest
Pulmonary Nodule Detection Using Deep Learning Technique	Outcome not relate to interest

Validation of a deep learning computer aided system for CT based lung nodule detection, classification, and growth rate estimation in a routine clinical population	Outcome not relate to interest
An Effective Approach for Automated Lung Node Detection using CT Scans	Outcome not relate to interest
Automated Computer-Aided Detection of Lung Nodules in Metastatic Colorectal Cancer Patients for the Identification of Pulmonary Oligometastatic Disease	Outcome not relate to interest
Improved Lung Cancer Detection in Ultra Low dose CT with Combined AI-based Nodule Detection and Denoising Techniques	Outcome not relate to interest
Deep Learning-based Artificial Intelligence Improves Accuracy of Error-prone Lung Nodules	Outcome not relate to interest
Deep Learning-Based Cancerous Lung Nodule Detection in Computed Tomography Imageries	Outcome not relate to interest
Cloud-Based Lung Tumor Detection and Stage Classification Using Deep Learning Techniques	Outcome not relate to interest
Comparison of two reader modes of computer-aided diagnosis in lung nodules on low-dose chest CT scan	Outcome not relate to interest
Clinical validation of deep learning algorithms for radiotherapy targeting of non-small-cell lung cancer: an observational study	Outcome not relate to interest
Higher agreement between readers with deep learning CAD software for reporting pulmonary nodules on CT	Outcome not relate to interest
Artificial intelligence supporting lung cancer screening: Computer aided diagnosis of lung lesions driven by morphological feature extraction	Outcome not relate to interest
A novel receptive field-regularized V-net and nodule classification network for lung nodule detection	Outcome not relate to interest
Detection and Stage Classification of UNet Segmented Lung Nodules Using CNN	Outcome not relate to interest
Diagnosis of Pulmonary Nodules using Multi-Size Multi-Branch 3D-CNN Architecture	Incomplete dataset specification, patient and nodule count duringn dataset splitting unclear
Clinical impact of a deep learning system for automated detection of missed pulmonary nodules on routine body computed tomography including the chest region	Outcome not relate to interest
Spatial Pyramid Pooling With 3D Convolution Improves Lung Cancer Detection	Outcome not relate to interest
LUNG CANCER IDENTIFICATION VIA DEEP LEARNING: A MULTI-STAGE WORKFLOW	Outcome not relate to interest
An enhanced UNet variant for Effective Lung Cancer Detection	Outcome not relate to interest
Artificial Algae Algorithm with Deep Transfer Learning based Lung Nodule Detection and Classification Model using CT images	Outcome not relate to interest
Diagnosis of Pulmonary Nodules using Multi-Size Multi-Branch 3D-CNN Architecture	Incomplete dataset specification, patient and nodule count duringn dataset splitting unclear
P42.06 Automatic Lung Nodule Detection by a Deep Learning-Based CAD System: The Value of Slab Thickness in the Maximum Intensity Projection Technique	Outcome not relate to interest
3D Multi-Branch Encoder-Decoder Networks with Attentional Feature Fusion for Pulmonary Nodule Detection in CT Scans	Conference published as original article later
Computed Tomography Image under Convolutional Neural Network Deep Learning Algorithm in Pulmonary Nodule Detection and Lung Function Examination	Outcome not relate to interest
An efficient multi-path 3D convolutional neural network for false-positive reduction of pulmonary nodule detection	False positive reduction task
Use of a Dual Artificial Intelligence Platform to Detect Unreported Lung Nodules	Outcome not relate to interest
Leveraging Auxiliary Information from EMR for Weakly Supervised Pulmonary Nodule Detection	Outcome not relate to interest
Diagnostic performance for pulmonary adenocarcinoma on CT: comparison of radiologists with and without three-dimensional convolutional neural network	Outcome not relate to interest
Dual Skip Connections Minimize the False Positive Rate of Lung Nodule Detection in CT images	Incomplete dataset specification, patient and nodule count duringn dataset splitting unclear
Automatic Localization of Lung Opacity in Chest CT Images - A Real-World Study	Outcome not relate to interest
A cascade and heterogeneous neural network for CT pulmonary nodule detection and its evaluation on both phantom and patient data	Outcome not relate to interest
Attribute prediction of concurrent nodules in lung CT in patients with lung adenocarcinoma using three-dimensional convolutional neural network	Outcome not relate to interest
IMAL-Net: Interpretable multi-task attention learning network for invasive lung adenocarcinoma screening in CT images	Outcome not relate to interest
Nodule Detection with Convolutional Neural Network Using Apache Spark and GPU Frameworks	Outcome not relate to interest
Attention-embedded complementary-stream CNN for false positive reduction in pulmonary nodule detection	False positive reduction task
Lung Nodule Detection based on Faster R-CNN Framework	Outcome not relate to interest

DFD-Net: lung cancer detection from denoised CT scan image using deep learning	Outcome not relate to interest
Lung Nodule Detection through CT Scan Images and DNN Models	Outcome not relate to interest
Size-Adaptive Deep Neural Networks Based Pulmonary Nodule Detection in CT Scans	Outcome not relate to interest
Can a Novel Deep Neural Network Improve the Computer-Aided Detection of Solid Pulmonary Nodules and the Rate of False-Positive Findings in Comparison to an Established Machine Learning Computer-Aided Detection?	Outcome not relate to interest
Computer-aided Detection of Subsolid Nodules at Chest CT: Improved Performance with Deep Learning-based CT Section Thickness Reduction	Outcome not relate to interest
Case Study of the Usefulness of Lung Nodule Detection Using Artificial Intelligence in Patient with Interstitial Pneumonia	Case
3D CNN with Visual Insights for Early Detection of Lung Cancer Using Gradient-Weighted Class Activation	Outcome not relate to interest
Multi-view Convolutional Recurrent Neural Networks for Lung Cancer Nodule Identification	Outcome not relate to interest
Multiscale CNN with compound fusions for false positive reduction in lung nodule detection	False positive reduction task
Clinical evaluation of a deep-learning-based computer-aided detection system for the detection of pulmonary nodules in a large teaching hospital	Outcome not relate to interest
Reducing False-Positives in Lung Nodules Detection Using Balanced Datasets	Outcome not relate to interest
Evaluation of the Effectiveness of Artificial Intelligence Chest CT Lung Nodule Detection Based on Deep Learning	Retracted
An Integration of blockchain and AI for secure data sharing and detection of CT images for the hospitals	Outcome not relate to interest
Detection of Overlooked Pulmonary Metastases in Serial CT Scans through Deep Learning-based Tracking of Longitudinal Changes	Outcome not relate to interest
Deep learning for lung cancer detection on screening ct scans: Results of a large-scale public competition and an observer study with 11 radiologists	Outcome not relate to interest
Lung cancer prediction by Deep Learning to identify benign lung nodules	Outcome not relate to interest
A comparison of the fusion model of deep learning neural networks with human observation for lung nodule detection and classification	Outcome not relate to interest
On the performance of lung nodule detection, segmentation and classification	Review
Analysis of lung cancer clinical diagnosis based on nodule detection from computed tomography images	Outcome not relate to interest
Evaluation of deep learning techniques for the detection of pulmonary nodules in computer tomography scans	Outcome not relate to interest
Nodules Detection by Deep Convolutional Neural Network and Its Application	Outcome not relate to interest
LNCDS: A 2D-3D cascaded CNN approach for lung nodule classification, detection and segmentation	Outcome not relate to interest
P42.02 Evaluating the Feasibility of a Deep Learning-Based Computer-Aided Detection System for Lung Nodule Detection in a Lung Cancer Screening Program	Outcome not relate to interest
Automatic detect lung node with deep learning in segmentation and imbalance data labeling	Outcome not relate to interest
High precision localization of pulmonary nodules on chest CT utilizing axial slice number labels	Outcome not relate to interest
Automated detection of lung nodules and coronary artery calcium using artificial intelligence on low-dose CT scans for lung cancer screening: accuracy and prognostic value	Outcome not relate to interest
Effect of CT reconstruction settings on the performance of a deep learning based lung nodule CAD system	Outcome not relate to interest
Validation of lung nodule detection a year before diagnosis in NLST dataset based on a deep learning system	Outcome not relate to interest
An Embedded Multi-branch 3D Convolution Neural Network for False Positive Reduction in Lung Nodule Detection	False positive reduction task
MR-Forest: A Deep Decision Framework for False Positive Reduction in Pulmonary Nodule Detection	Not deep learning
Effect of slab thickness on pulmonary nodule detection using maximum intensity projection in a deep learning-based computer-aided detection system	Outcome not relate to interest
Design of Automatic Lung Nodule Detection System Based on Multi-Scene Deep Learning Framework	Outcome not relate to interest
Deep Learning Assisted Predict of Lung Cancer on Computed Tomography Images Using the Adaptive Hierarchical Heuristic Mathematical Model	Outcome not relate to interest
Pulmonary Nodule Detection Using V-Net and High-Level Descriptor Based SVM Classifier	Outcome not relate to interest
MD-NDNet: a multi-dimensional convolutional neural network for false-positive reduction in pulmonary nodule detection	False positive reduction task
Evaluating Deep Learning Algorithms in Pulmonary Nodule Detection()	Outcome not relate to interest
Automated lung nodule candidate detection using an iteratively optimized multi-resolution 3d depthwise separable cnns with effective training initialization	Outcome not relate to interest

A robust convolutional neural network for lung nodule detection in the presence of foreign bodies	Not CT
Automated Decision Support System for Lung Cancer Detection and Classification via Enhanced RFCN With Multilayer Fusion RPN	Outcome not relate to interest
Multi-level 3d densenets for false-positive reduction in lung nodule detection based on chest computed tomography	False positive reduction task
DeepSEED: 3D Squeeze-and-Excitation Encoder-Decoder Convolutional Neural Networks for Pulmonary Nodule Detection	Conference
Study on the Detection of Pulmonary Nodules in CT Images Based on Deep Learning	Outcome not relate to interest
FALSE POSITIVE REDUCTION BASED ON ANATOMICAL CHARACTERIZATION USING DEEP LEARNING NEURAL NETWORK IN LUNG NODULE DETECTION	Outcome not relate to interest
Efficiency of a computer-aided diagnosis (CAD) system with deep learning in detection of pulmonary nodules on 1-mm-thick images of computed tomography	Outcome not relate to interest
Diagnosis of lung nodules from 2D computer tomography scans	Outcome not relate to interest
Deep-learning-based model observer for a lung nodule detection task in computed tomography	Outcome not relate to interest
Recurrent attention network for false positive reduction in the detection of pulmonary nodules in thoracic CT scans	False positive reduction task
Multi-view Convolutional Neural Network for lung nodule false positive reduction	Outcome not relate to interest
A two-stage framework for automated malignant pulmonary nodule detection in CT scans	Outcome not relate to interest
Validation of a deep learning-based computer-aided system for lung nodule detection in a Chinese lung cancer screening program	Outcome not relate to interest
Development and clinical application of deep learning model for lung nodules screening on CT images	Outcome not relate to interest
Incidence lung cancer after a negative ct screening in the national lung screening trial: Deep learning-based detection of missed lung cancers	Outcome not relate to interest
A Novel Pulmonary Nodule Detection Model Based on Multi-Step Cascaded Networks	Outcome not relate to interest
Deep Learning for the Classification of Small (≤2 cm) Pulmonary Nodules on CT Imaging: A Preliminary Study	Outcome not relate to interest
Deep learning for lung Cancer detection and classification	Outcome not relate to interest
Automatic Lung Nodule Detection Combined with Gaze Information Improves Radiologists' Screening Performance	Outcome not relate to interest
An Automatic Lung Cancer Detection and Classification (ALCDC) System Using Convolutional Neural Network	Outcome not relate to interest
Automatic classification of lung nodule candidates based on a novel 3D convolution network and knowledge transferred from a 2D network	False positive reduction task
Deep CNN models for pulmonary nodule classification: Model modification, model integration, and transfer learning	Outcome not relate to interest
A Pulmonary Nodule Detection Algorithm Based on Low Dose CT Images	Outcome not relate to interest
Multi-Scale Heterogeneous 3D CNN for False-Positive Reduction in Pulmonary Nodule Detection, Based on Chest CT Images	False positive reduction task
Lung Nodule Detection in CT Images Using a Raw Patch-Based Convolutional Neural Network	Outcome not relate to interest
Lung Cancer Detection using Co-learning from Chest CT Images and Clinical Demographics	Outcome not relate to interest
Research progress on computed tomography image detection and classification of pulmonary nodule based on deep learning	Review
Expert knowledge-infused deep learning for automatic lung nodule detection	Outcome not relate to interest
Multi-path convolutional neural network for lung cancer detection	Outcome not relate to interest
Automatic Lung Nodule Detection in CT Images Using Convolutional Neural Networks	Outcome not relate to interest
Analysis of Deep Convolutional Features for Detection of Lung Nodules in Computed Tomography	Outcome not relate to interest
Lung Cancer Detection Based on CT Scan Images by Using Deep Transfer Learning	Outcome not relate to interest
Lung Nodule Detection based on Ensemble of Hand Crafted and Deep Features	Outcome not relate to interest
3-D Convolutional Neural Networks for Automatic Detection of Pulmonary Nodules in Chest CT	Outcome not relate to interest
A 3D Lung Nodule Candidate Detection by Grouping DCNN 2D Candidates	Outcome not relate to interest
Automated detection and segmentation of lung tumors using deep learning	Outcome not relate to interest
Automated detection and classification for early stage lung cancer on CT images using deep learning	Outcome not relate to interest
Relu Cascade of Feature Pyramid Networks for CT Pulmonary Nodule Detection	Outcome not relate to interest
Ensemble Learning of Multiple-View 3D-CNNs Model for Micro-Nodules Identification in CT images	Outcome not relate to interest
Lung Tumor Classification and Detection from CT Scan Images using Deep Convolutional Neural Networks (DCNN)	Outcome not relate to interest

Automatic Computer Aided System for Lung Cancer in Chest CTs Using MD-RFCN Combined with Tri-Level Region Proposal Network	Outcome not relate to interest
Automatic detection and diagnosis of pulmonary nodule using deep convolutional neural network	Outcome not relate to interest
[Pulmonary nodule detection method based on convolutional neural network]	Outcome not relate to interest
Deep learning analysis for automatic lung nodule detection	Outcome not relate to interest
Evaluating a fully automated pulmonary nodule detection approach and its impact on radiologist performance	Outcome not relate to interest
3DFPN-HS²: 3D Feature Pyramid Network Based High Sensitivity and Specificity Pulmonary Nodule Detection	Conference published as original article later
Deep learning-based cad may improve detection of pulmonary nodules while preserving a low false-positive rate	Outcome not relate to interest
Lung Nodule Detection With Deep Learning in 3D Thoracic MR Images	Outcome not relate to interest
YOLO V2 network with asymmetric convolution kernel for lung nodule detection of CT image	Outcome not relate to interest
Performance of deep-learning-based artificial intelligence on detection of pulmonary nodules in chest CT	Outcome not relate to interest
Evaluating the performance of a deep learning-based computer-aided diagnosis (DL-CAD) system for detecting and characterizing lung nodules: Comparison with the performance of double reading by radiologists	Outcome not relate to interest
LUNG NODULE DETECTION WITH A 3D CONVNET VIA IOU SELF-NORMALIZATION AND MAXOUT UNIT	Conference
Detection of Lung Nodules Using Unsupervised Machine Learning Method	Outcome not relate to interest
Multi-scale gradual integration CNN for false positive reduction in pulmonary nodule detection	False positive reduction task
Lung Cancer Detection from Computed Tomography (CT) Scans using Convolutional Neural Network	Outcome not relate to interest
A collaborative computer aided diagnosis (C-CAD) system with eye-tracking, sparse attentional model, and deep learning	Outcome not relate to interest
Pulmonary nodule detection on computed tomography using neuro-evolutionary scheme	Outcome not relate to interest
Volume Visualization for Improving CT Lung Nodule Detection	Outcome not relate to interest
Identification of pulmonary nodules via CT images with hierarchical fully convolutional networks	Outcome not relate to interest
Automatic Detection and Segmentation of Lung Lesions using Deep Residual CNNs	Outcome not relate to interest
A multimodal neural network for lung nodule detection with low-dose CT images	Outcome not relate to interest
End-to-end lung cancer screening with three-dimensional deep learning on low-dose chest computed tomography (vol 25, pg 954, 2019)	Outcome not relate to interest
DeepLung: Deep 3D Dual Path Nets for Automated Pulmonary Nodule Detection and Classification	Conference
A deep-learning based automatic pulmonary nodule detection system	Outcome not relate to interest
Lung Nodule Detection via 3D U-Net and Contextual Convolutional Neural Network	Outcome not relate to interest
Lung Nodule Detection Using Combined Traditional and Deep Models and Chest CT	Conference
DeepLesion: Automated mining of large-scale lesion annotations and universal lesion detection with deep learning	Outcome not relate to interest
Lung Cancer Detection and Classification using Deep Learning	Outcome not relate to interest
AUTOMATED PULMONARY NODULE DETECTION USING 3D DEEP CONVOLUTIONAL NEURAL NETWORKS	Conference
A Fast Automatic Juxta-pleural Lung Nodule Detection Framework Using Convolutional Neural Networks and Vote Algorithm	Outcome not relate to interest
Deep Convolutional Nets for Pulmonary Nodule Detection and Classification	Conference
A Generalized Deep Learning-Based Diagnostic System for Early Diagnosis of Various Types of Pulmonary Nodules	Outcome not relate to interest
Using YOLO based deep learning network for real time detection and localization of lung nodules from low dose CT scans	Outcome not relate to interest
Effect of Input Size on the Classification of Lung Nodules Using Convolutional Neural Networks	Not english
Classification of lung nodules in CT scans using three-dimensional deep convolutional neural networks with a checkpoint ensemble method	False positive reduction task
A deep 3D residual CNN for false-positive reduction in pulmonary nodule detection	False positive reduction task
An Automatic Detection System of Lung Nodule Based on Multigroup Patch-Based Deep Learning Network	Outcome not relate to interest
Lung nodule detection from CT scans using 3D convolutional neural networks without candidate selection	Outcome not relate to interest
Deep Learning for Lung Lesion Detection	Outcome not relate to interest

3D deep learning for detecting pulmonary nodules in CT scans	Outcome not relate to interest
A Novel Computer-Aided Lung Cancer Detection Method Based on Transfer Learning from GoogLeNet and Median Intensity Projections	Outcome not relate to interest
Single-view 2D CNNs with fully automatic non-nodule categorization for false positive reduction in pulmonary nodule detection	False positive reduction task
Automated Detection of Lung Nodules in CT Images with 3D Convolutional Neural Networks	Outcome not relate to interest
Convolutional neural network-based PSO for lung nodule false positive reduction on CT images	Outcome not relate to interest
3D Convolutional Neural Networks Fusion Model for Lung Nodule Detection on Clinical CT Scans	Conference
Towards an Automatic Lung Cancer Screening System in Low Dose Computed Tomography	Outcome not relate to interest
Lung nodule detection via deep reinforcement learning	Outcome not relate to interest
Deep learning system for lung nodule detection	Outcome not relate to interest
Automated Detection of Lung Nodules with Three-dimensional Convolutional Neural Networks	Outcome not relate to interest
Deep Learning Based Nodule Detection from Pulmonary CT Images	Outcome not relate to interest
Automatic Detection of Lung Nodules: False positive reduction using convolution neural networks and handcrafted features	Outcome not relate to interest
A Novel Fusion Approach for Early Lung Cancer Detection Using Computer Aided Diagnosis Techniques	Outcome not relate to interest
Lung Nodule Detection Based on 3D Convolutional Neural Networks	Outcome not relate to interest
Multilevel Contextual 3-D CNNs for False Positive Reduction in Pulmonary Nodule Detection	False positive reduction task
Lung Cancer Detection: A Deep Learning Approach	Outcome not relate to interest
Lung cancer detection and classification with 3D convolutional neural network (3D-CNN)	Outcome not relate to interest
Pulmonary Nodule Detection in CT Images: False Positive Reduction Using Multi-View Convolutional Networks	Outcome not relate to interest
Convolutional Neural Networks for Lung Cancer Screening in Computed Tomography (CT) Scans	Outcome not relate to interest
Pulmonary Nodule Classification with Deep Convolutional Neural Networks on Computed Tomography Images	Outcome not relate to interest
Lung Nodule Detection in CT Images using Deep Convolutional Neural Networks	Outcome not relate to interest

Table S5. CT Protocol and hardware characteristics

First author	In-plane resolution	Slice thickness	Hardware
Zhao et al. (2023) [29]	I: 0.69 (0.46–0.98); G: 0.78(0.58–0.98) -mean	I: 1.57 (0.45–2.50) ; E: 5 (1.00–5.34) -mean	seven different GE Medical Systems LightSpeed scanner models, four different Philips Brilliance scanner models, five different Siemens Definition, Emotion, and Sensation scanner models, Toshiba Aquilion scanners
Zhang et al. (2021) [30]	0.69 (0.46–0.98) -mean	1.57 (0.45–2.50) mean	seven different GE Medical Systems LightSpeed scanner models, four different Philips Brilliance scanner models, five different Siemens Definition, Emotion, and Sensation scanner models, Toshiba Aquilion scanners
Zhang et al. (2023) [3-1]	I: 0.69 (0.46–0.98) - mean	I: 1.57 (0.45–2.50) -mean	seven different GE Medical Systems LightSpeed scanner models, four different Philips Brilliance scanner models, five different Siemens Definition, Emotion, and Sensation scanner models, Toshiba Aquilion scanners
Xu et al. (2023) [32]	I1 (0.64~0.86); I2 (0.66~0.69); I3: (0.62~0.78); I4: (1~1)	I1: (0.5~2.5); I2: (0.3~2.5); I3: (0.4~0.8); I4: (1~1)	I1: seven different GE Medical Systems LightSpeed scanner models, four different Philips Brilliance scanner models, five different Siemens Definition, Emotion, and Sensation scanner models, Toshiba Aquilion scanners; I4: 2,652 scans from ten different GE Medical Systems scanner models, 2,305 scans from eleven different Siemens scanner models, 2,224 scans from three different Toshiba scanner models, 800 scans from two different United Imaging Healthcare (UIH) scanner models, and 817 scans from six different Philips scanner models
Wang et al. (2023) [33]	0.69 (0.46–0.98) -mean	1.57 (0.45–2.50) mean	seven different GE Medical Systems LightSpeed scanner models, four different Philips Brilliance scanner models, five different Siemens Definition, Emotion, and Sensation scanner models, Toshiba Aquilion scanners
Shen et al. (2023) [34]	0.69 (0.46–0.98) -mean	1.57 (0.45–2.50) mean	seven different GE Medical Systems LightSpeed scanner models, four different Philips Brilliance scanner models, five different Siemens Definition, Emotion, and Sensation scanner models, Toshiba Aquilion scanners
Mkindu et al. (2023) I [35]	0.69 (0.46–0.98) -mean	1.57 (0.45–2.50) mean	seven different GE Medical Systems LightSpeed scanner models, four different Philips Brilliance scanner models, five different Siemens Definition, Emotion, and Sensation scanner models, Toshiba Aquilion scanners

Mkindu et al. (2023) II [36]	0.69 (0.46–0.98) -mean	1.57 (0.45–2.50) mean	seven different GE Medical Systems LightSpeed scanner models, four different Philips Brilliance scanner models, five different Siemens Definition, Emotion, and Sensation scanner models, Toshiba Aquilion scanners
Lin et al. (2023) [37]	0.69 (0.46–0.98) -mean	1.57 (0.45–2.50) mean	seven different GE Medical Systems LightSpeed scanner models, four different Philips Brilliance scanner models, five different Siemens Definition, Emotion, and Sensation scanner models, Toshiba Aquilion scanners
Hendrix et al. (2023) [38]	NR	NR	Canon Aquilion One; Canon Aquilion Precision; Canon Aquilion CXL; GE LightSpeed 16; GE LightSpeed Ultra; GE LightSpeed QX/I; GE LightSpeed pro 16; GE LightSpeed VCT; GE LightSpeed Plus; Philips Brilliance 16P; Siemens Sensation 16; Siemens Sensation 64; Other; I test: Canon Aquilion One; Canon Aquilion Precision; Canon Aquilion CXL; Philips Brilliance iCT 256; Siemens Biograph 40; Other E: Siemens Somatom Definition Flash; Siemens Somatom Definition AS+; Siemens Somatom Edge; Siemens Sensation 64; Other
Guo et al. (2023) [39]	NR	I1: 5mm/ I2: 1mm	NR
Chen et al. (2023) [40]	0.69 (0.46–0.98) -mean	1.57 (0.45–2.50) mean	seven different GE Medical Systems LightSpeed scanner models, four different Philips Brilliance scanner models, five different Siemens Definition, Emotion, and Sensation scanner models, Toshiba Aquilion scanners
Zheng et al. (2022) [41]	0.69 (0.46–0.98) -mean	1.57 (0.45–2.50) mean	seven different GE Medical Systems LightSpeed scanner models, four different Philips Brilliance scanner models, five different Siemens Definition, Emotion, and Sensation scanner models, Toshiba Aquilion scanners
Hui Zhang et al. (2022) [42]	I1 (0.64~0.86); I2 (0.66~0.69);	I1: (0.5~2.5); I2: (0.3~2.5);	seven different GE Medical Systems LightSpeed scanner models, four different Philips Brilliance scanner models, five different Siemens Definition, Emotion, and Sensation scanner models, Toshiba Aquilion scanners
Guanglu Zhang et al. (2022) [43]	0.69 (0.46–0.98) -mean	1.57 (0.45–2.50) mean	seven different GE Medical Systems LightSpeed scanner models, four different Philips Brilliance scanner models, five different Siemens Definition, Emotion, and Sensation scanner models, Toshiba Aquilion scanners
Yuan et al. (2022) [44]	0.69 (0.46–0.98) -mean	1.57 (0.45–2.50) mean	seven different GE Medical Systems LightSpeed scanner models, four different Philips Brilliance scanner models, five different Siemens Definition, Emotion, and Sensation scanner models, Toshiba Aquilion scanners
Suzuki et al. (2022) [45]	0.69 (0.46–0.98) -mean	1.57 (0.45–2.50) mean	seven different GE Medical Systems LightSpeed scanner models, four different Philips Brilliance scanner models, five different Siemens Definition, Emotion, and Sensation scanner models, Toshiba Aquilion scanners
Niu et al.(2022) [46]	0.69 (0.46–0.98) -mean	1.57 (0.45–2.50) mean	seven different GE Medical Systems LightSpeed scanner models, four different Philips Brilliance scanner models, five different Siemens Definition, Emotion, and Sensation scanner models, Toshiba Aquilion scanners
Mei et al. (2022) [47]	0.706(0.310~1.091) -mean	(0.4~2.5)	2,652 scans from ten different GE Medical Systems scanner models, 2,305 scans from eleven different Siemens scanner models, 2,224 scans from three different Toshiba scanner models, 800 scans from two different United Imaging Healthcare (UIH) scanner models, and 817 scans from six different Philips scanner models
Ma et al. (2022) [48]	0.7 -mean	0.92 -mean	SIEMENS; TOSHIBA; GE; Philips
Luo et al. (2022) [49]	0.69 (0.46–0.98) -mean	1.57 (0.45–2.50) mean	seven different GE Medical Systems LightSpeed scanner models, four different Philips Brilliance scanner models, five different Siemens Definition, Emotion, and Sensation scanner models, Toshiba Aquilion scanners
Liu et al. (2022) [50]	I: 0.69 (0.46–0.98); E1: 0.685(0.54-0.9); E2:0.58 -mean	I: 1.57 (0.45–2.50); E1: 1; E2: 5-mean	seven different GE Medical Systems LightSpeed scanner models, four different Philips Brilliance scanner models, five different Siemens Definition, Emotion, and Sensation scanner models, Toshiba Aquilion scanners
Katase et al. (2022) [51]	E1: 0.685(0.54-0.9); E2: 0.63	E1: 1 E2: 1(0.5-1) -mean	Toshiba Medical Systems Corp, GE Healthcare, Siemens Healthineers AG,Philips
Huang et al. (2022) [52]	0.69 (0.46–0.98) -mean	1.57 (0.45–2.50) mean	seven different GE Medical Systems LightSpeed scanner models, four different Philips Brilliance scanner models, five different Siemens Definition, Emotion, and Sensation scanner models, Toshiba Aquilion scanners
Guo et al. (2022) [53]	0.69 (0.46–0.98) -mean	1.57 (0.45–2.50) mean	seven different GE Medical Systems LightSpeed scanner models, four different Philips Brilliance scanner models, five different Siemens Definition, Emotion, and Sensation scanner models, Toshiba Aquilion scanners
Pereira et al. (2021) [54]	0.69 (0.46–0.98) -mean	1.57 (0.45–2.50) mean	seven different GE Medical Systems LightSpeed scanner models, four different Philips Brilliance scanner models, five different Siemens Definition, Emotion, and Sensation scanner models, Toshiba Aquilion scanners
Peng et al. (2021) [55]	0.69 (0.46–0.98) -mean	1.57 (0.45–2.50) mean	seven different GE Medical Systems LightSpeed scanner models, four different Philips Brilliance scanner models, five different Siemens Definition, Emotion, and Sensation scanner models, Toshiba Aquilion scanners

Nguyen et al. (2021) [56]	0.69 (0.46–0.98) -mean	1.57 (0.45–2.50) mean	seven different GE Medical Systems LightSpeed scanner models, four different Philips Brilliance scanner models, five different Siemens Definition, Emotion, and Sensation scanner models, Toshiba Aquilion scanners
Weihua Liu et al. (2021) [57]	0.69 (0.46–0.98) -mean	1.57 (0.45–2.50) mean	seven different GE Medical Systems LightSpeed scanner models, four different Philips Brilliance scanner models, five different Siemens Definition, Emotion, and Sensation scanner models, Toshiba Aquilion scanners
Siqi Liu et al. (2021) [58]	NR	NR	seven different GE Medical Systems LightSpeed scanner models, four different Philips Brilliance scanner models, five different Siemens Definition, Emotion, and Sensation scanner models, Toshiba Aquilion scanners
Farhangi et al. (2021) [59]	0.69 (0.46–0.98) -mean	1.57 (0.45–2.50) mean	seven different GE Medical Systems LightSpeed scanner models, four different Philips Brilliance scanner models, five different Siemens Definition, Emotion, and Sensation scanner models, Toshiba Aquilion scanners
Zheng et al. (2020) [60]	0.69 (0.46–0.98) -mean	1.57 (0.45–2.50) mean	seven different GE Medical Systems LightSpeed scanner models, four different Philips Brilliance scanner models, five different Siemens Definition, Emotion, and Sensation scanner models, Toshiba Aquilion scanners
Xu et al. (2020) [61]	NR	1	Siemens Definition AS+
Tan et al. (2020). [62]	I: 0.69 (0.46–0.98) ; E: NR (0.62-0.94)-mean	I: 1.57 (0.45–2.50);E: NR(1.5~2) mean	seven different GE Medical Systems LightSpeed scanner models, four different Philips Brilliance scanner models, five different Siemens Definition, Emotion, and Sensation scanner models, Toshiba Aquilion scanners
Ozdemir et al. (2020) [63]	0.69 (0.46–0.98) -mean	1.57 (0.45–2.50) mean	seven different GE Medical Systems LightSpeed scanner models, four different Philips Brilliance scanner models, five different Siemens Definition, Emotion, and Sensation scanner models, Toshiba Aquilion scanners
Masood et al. (2020) [64]	0.69 (0.46–0.98) -mean	1.57 (0.45–2.50) mean	seven different GE Medical Systems LightSpeed scanner models, four different Philips Brilliance scanner models, five different Siemens Definition, Emotion, and Sensation scanner models, Toshiba Aquilion scanners
Guo et al. (2020) [65]	NR	I1: 5mm/ I2: 1.5 mm	NR
Chenyang et al. (2020) [66]	0.69 (0.46–0.98) -mean	1.57 (0.45–2.50) mean	seven different GE Medical Systems LightSpeed scanner models, four different Philips Brilliance scanner models, five different Siemens Definition, Emotion, and Sensation scanner models, Toshiba Aquilion scanners
Cao et al. (2020) [67]	0.69 (0.46–0.98) -mean	1.57 (0.45–2.50) mean	seven different GE Medical Systems LightSpeed scanner models, four different Philips Brilliance scanner models, five different Siemens Definition, Emotion, and Sensation scanner models, Toshiba Aquilion scanners
Xie et al. (2019) [68]	0.69 (0.46–0.98) -mean	1.57 (0.45–2.50) mean	seven different GE Medical Systems LightSpeed scanner models, four different Philips Brilliance scanner models, five different Siemens Definition, Emotion, and Sensation scanner models, Toshiba Aquilion scanners
Winkels et al. (2019) [69]	E: 0.69 (0.46–0.98) -mean	E: 1.57 (0.45–2.50) mean	
Nasrullah et al. (2019) [70]	0.69 (0.46–0.98) -mean	1.57 (0.45–2.50) mean	seven different GE Medical Systems LightSpeed scanner models, four different Philips Brilliance scanner models, five different Siemens Definition, Emotion, and Sensation scanner models, Toshiba Aquilion scanners
Xia Huang et al. (2019) [71]	0.69 (0.46–0.98) -mean	1.57 (0.45–2.50) mean	seven different GE Medical Systems LightSpeed scanner models, four different Philips Brilliance scanner models, five different Siemens Definition, Emotion, and Sensation scanner models, Toshiba Aquilion scanners
Wenkai Huang et al. (2019) [72]	NR	NR	
Gong et al. (2019) [73]	0.69 (0.46–0.98) -mean	1.57 (0.45–2.50) mean	seven different GE Medical Systems LightSpeed scanner models, four different Philips Brilliance scanner models, five different Siemens Definition, Emotion, and Sensation scanner models, Toshiba Aquilion scanners
Zhang et al. (2018) [74]	0.69 (0.46–0.98) -mean	1.57 (0.45–2.50) mean	seven different GE Medical Systems LightSpeed scanner models, four different Philips Brilliance scanner models, five different Siemens Definition, Emotion, and Sensation scanner models, Toshiba Aquilion scanners
Gu et al. (2018) [75]	0.69 (0.46–0.98) -mean	1.57 (0.45–2.50) mean	seven different GE Medical Systems LightSpeed scanner models, four different Philips Brilliance scanner models, five different Siemens Definition, Emotion, and Sensation scanner models, Toshiba Aquilion scanners
Setio et al. (2018) [76]	0.69 (0.46–0.98) -mean	1.57 (0.45–2.50) mean	seven different GE Medical Systems LightSpeed scanner models, four different Philips Brilliance scanner models, five different Siemens Definition, Emotion, and Sensation scanner models, Toshiba Aquilion scanners

Table S6. Quality assessment according to the Quality Assessment of Diagnostic Accuracy Studies 2 (QUADAS-2) criteria

Source	Risk of bias											Concern of applicability		
	Patient selection:			INDEX TEST		Reference Standard		Flow and Timing						
	Consecutive	Case-control	Inappropriate exclusions	Blind to reference standard	Threshold prespecified	Correctly classify the target condition	Blind to index test	Appropriate interval	Receive a reference standard	Same reference standard	All patients analyzed	Patient selection	INDEX TEST	Reference Standard
Zhao et al. (2023) [29]	Yes	No	No	Yes	Yes	Yes	Yes	Yes	Yes	Yes	Yes	Low	Low	Low
Zhang et al. (2021) [30]	Unclear	No	No	Yes	Yes	Yes	Yes	Yes	Yes	Yes	Yes	Low	Low	Low
Zhang et al. (2023) [31]	Unclear	No	No	Yes	Yes	Yes	Yes	Yes	Yes	Yes	Yes	Low	Low	Low
Xu et al. (2023) [32]	Yes	No	No	Yes	Yes	Yes	Yes	Yes	Yes	Yes	Yes	Low	Low	Low
Wang et al. (2023) [33]	Unclear	No	No	Yes	Yes	Yes	Yes	Yes	Yes	Yes	Yes	Low	Low	Low
Shen et al. (2023) [34]	Unclear	No	No	Yes	Yes	Yes	Yes	Yes	Yes	Yes	Yes	Low	Low	Low
Mkindu et al. (2023) I [35]	Unclear	No	No	Yes	Yes	Yes	Yes	Yes	Yes	Yes	Yes	Low	Low	Low
Mkindu et al. (2023) II [36]	Unclear	No	No	Yes	Yes	Yes	Yes	Yes	Yes	Yes	Yes	Low	Low	Low
Lin et al. (2023) [37]	Unclear	No	No	Yes	Yes	Yes	Yes	Yes	Yes	Yes	Yes	Low	Low	Low
Hendrix et al. (2023) [38]	Yes	No	No	Yes	Yes	Yes	Yes	Yes	Yes	Yes	Yes	Low	Low	Low
Guo et al. (2023) [39]	Yes	No	Unclear	Yes	Yes	Yes	Yes	Yes	Yes	Yes	Yes	Low	Low	Low
Chen et al. (2023) [40]	Unclear	No	No	Yes	Yes	Yes	Yes	Yes	Yes	Yes	Yes	Low	Low	Low
Zheng et al. (2022) [41]	Unclear	No	No	Yes	Yes	Yes	Yes	Yes	Yes	Yes	Yes	Low	Low	Low
Hui Zhang et al. (2022) [42]	Unclear	No	No	Yes	Yes	Yes	Yes	Yes	Yes	Yes	Yes	Low	Low	Low
Guanglu Zhang et al. (2022) [43]	Unclear	No	No	Yes	Yes	Yes	Yes	Yes	Yes	Yes	Yes	Low	Low	Low
Yuan et al. (2022) [44]	Unclear	No	No	Yes	Yes	Yes	Yes	Yes	Yes	Yes	Yes	Low	Low	Low
Suzuki et al. (2022) [45]	Unclear	No	No	Yes	Yes	Yes	Yes	Yes	Yes	Yes	Yes	Low	Low	Low
Niu et al.(2022) [46]	Unclear	No	No	Yes	Yes	Yes	Yes	Yes	Yes	Yes	Yes	Low	Low	Low
Mei et al. (2022) [47]	Yes	No	No	Yes	Yes	Yes	Yes	Yes	Yes	Yes	Yes	Low	Low	Low
Ma et al. (2022) [48]	Unclear	No	No	Yes	Yes	Yes	Yes	Yes	Yes	Yes	Yes	Low	Low	Low
Luo et al. (2022) [49]	Unclear	No	No	Yes	Yes	Yes	Yes	Yes	Yes	Yes	Yes	Low	Low	Low
Liu et al. (2022) [50]	Unclear	No	No	Yes	Yes	Yes	Yes	Yes	Yes	Yes	Yes	Low	Low	Low
Katase et al. (2022) [51]	Yes	No	No	Yes	Yes	Yes	Yes	Yes	Yes	Yes	No	Low	Low	Low

Huang et al. (2022) [52]	Unclear	No	No	Yes	Yes	Yes	Yes	Yes	Yes	Yes	Yes	Low	Low	Low
Guo et al. (2022) [53]	Unclear	No	No	Yes	Yes	Yes	Yes	Yes	Yes	Yes	Yes	Low	Low	Low
Pereira et al. (2021) [54]	Unclear	No	No	Yes	Yes	Yes	Yes	Yes	Yes	Yes	Yes	Low	Low	Low
Peng et al. (2021) [55]	Unclear	No	No	Yes	Yes	Yes	Yes	Yes	Yes	Yes	Yes	Low	Low	Low
Nguyen et al. (2021) [56]	Unclear	No	No	Yes	Yes	Yes	Yes	Yes	Yes	Yes	Yes	Low	Low	Low
Weihua Liu et al. (2021) [57]	Unclear	No	No	Yes	Yes	Yes	Yes	Yes	Yes	Yes	Yes	Low	Low	Low
Siqi Liu et al. (2021) [58]	Unclear	No	No	Yes	Yes	Yes	Yes	Yes	Yes	Yes	Yes	Low	Low	Low
Farhangi et al. (2021) [59]	Unclear	No	No	Yes	Yes	Yes	Yes	Yes	Yes	Yes	Yes	Low	Low	Low
Zheng et al. (2020) [60]	Unclear	No	No	Yes	Yes	Yes	Yes	Yes	Yes	Yes	Yes	Low	Low	Low
Xu et al. (2020) [61]	Yes	No	No	Yes	Yes	Yes	Yes	Yes	Yes	Yes	Yes	Low	Low	Low
Tan et al. (2020). [62]	Unclear	No	No	Yes	Yes	Yes	Yes	Yes	Yes	Yes	Yes	Low	Low	Low
Ozdemir et al. (2020) [63]	Unclear	No	No	Yes	Yes	Yes	Yes	Yes	Yes	Yes	Yes	Low	Low	Low
Masood et al. (2020) [64]	Unclear	No	No	Yes	Yes	Yes	Yes	Yes	Yes	Yes	Yes	Low	Low	Low
Guo et al. (2020) [65]	Unclear	No	No	Yes	Yes	Yes	Yes	Yes	Yes	Yes	Yes	Low	Low	Low
Chenyang et al. (2020) [66]	Unclear	No	No	Yes	Yes	Yes	Yes	Yes	Yes	Yes	Yes	Low	Low	Low
Cao et al. (2020) [67]	Unclear	No	No	Yes	Yes	Yes	Yes	Yes	Yes	Yes	Yes	Low	Low	Low
Xie et al. (2019) [68]	Unclear	No	No	Yes	Yes	Yes	Yes	Yes	Yes	Yes	Yes	Low	Low	Low
Winkels et al. (2019) [69]	Unclear	No	No	Yes	Yes	Yes	Yes	Yes	Yes	Yes	Yes	Low	Low	Low
Nasrullah et al. (2019) [70]	Unclear	No	No	Yes	Yes	Yes	Yes	Yes	Yes	Yes	Yes	Low	Low	Low
Xia Huang et al. (2019) [71]	Unclear	No	No	Yes	Yes	Yes	Yes	Yes	Yes	Yes	Yes	Low	Low	Low
Wenkai Huang et al. (2019) [72]	Unclear	No	No	Yes	Yes	Yes	Yes	Yes	Yes	Yes	Yes	Low	Low	Low
Gong et al. (2019) [73]	Unclear	No	No	Yes	Yes	Yes	Yes	Yes	Yes	Yes	Yes	Low	Low	Low
Zhang et al. (2018) [74]	Unclear	No	No	Yes	Yes	Yes	Yes	Yes	Yes	Yes	Yes	Low	Low	Low
Gu et al. (2018) [75]	Unclear	No	No	Yes	Yes	Yes	Yes	Yes	Yes	Yes	Yes	Low	Low	Low
Setio et al. (2018) [76]	Unclear	No	No	Yes	Yes	Yes	Yes	Yes	Yes	Yes	Yes	Low	Low	Low

Table S7. The Checklist for Artificial Intelligence in Medical Imaging scores.

Source	Title/Abstract	Introduction	Methods							Results		Discussion	Other Information	Total Score
			Study design	Data	Ground truth	Data preparation	Model	Training	Evaluation	Data	Model performance			
	(2)	(2)	(2)	(7)	(5)	(3)	(3)	(3)	(5)	(2)	(3)	(2)	(3)	(42)
Zhao et al. (2023) [29]	2	2	1	4	3	2	1	2	3	0	2	2	1	25
Zhang et al. (2021) [30]	2	2	1	5	3	2	1	0	3	0	1	0	1	21
Zhang et al. (2023) [31]	2	2	1	5	3	2	2	2	4	0	1	1	0	25
Xu et al. (2023) [32]	1	2	1	5	3	2	3	2	4	0	1	1	0	25
Wang et al. (2023) [33]	1	2	2	5	3	2	3	3	4	0	1	1	2	29
Shen et al. (2023) [34]	2	2	1	5	3	2	2	2	3	0	1	2	1	26
Mkindu et al. (2023) I [35]	2	2	1	5	3	2	1	1	3	0	1	1	1	23
Mkindu et al. (2023) II [36]	2	2	1	5	3	2	2	2	3	0	1	1	1	25
Lin et al. (2023) [37]	1	2	1	5	3	2	1	2	3	0	1	2	1	24
Hendrix et al. (2023) [38]	2	2	2	6	5	3	2	2	5	2	2	2	3	38
Guo et al. (2023) [39]	2	2	2	5	3	2	1	2	4	1	2	2	0	28
Chen et al. (2023) [40]	2	2	1	7	3	2	1	2	3	0	1	1	1	26
Zheng et al. (2022) [41]	2	2	1	5	3	2	2	2	4	0	1	1	1	26
Hui Zhang et al. (2022) [42]	1	2	1	4	2	2	2	2	3	0	1	1	1	22
Guanglu Zhang et al. (2022) [43]	2	2	1	5	3	2	2	1	3	0	1	2	1	25
Yuan et al. (2022) [44]	2	2	1	5	3	2	2	2	4	0	1	2	1	27
Suzuki et al. (2022) [45]	2	2	2	5	4	2	2	2	3	0	1	2	1	28
Niu et al.(2022) [46]	1	2	1	5	3	2	2	1	3	0	1	1	0	22
Mei et al. (2022) [47]	1	2	1	5	3	2	2	1	4	0	1	1	1	24
Ma et al. (2022) [48]	2	2	1	5	3	2	2	2	4	0	2	2	1	28
Luo et al. (2022) [49]	2	2	1	5	3	2	2	2	4	0	1	1	2	27
Liu et al. (2022) [50]	2	2	1	5	3	1	2	2	4	0	1	1	1	25
Katase et al. (2022) [51]	2	2	2	5	3	2	1	1	5	1	1	1	1	27
Huang et al. (2022) [52]	2	2	1	5	3	2	1	1	3	0	1	1	2	24

Guo et al. (2022) [53]	1	2	1	5	3	2	3	2	5	0	2	1	1	28
Pereira et al. (2021) [54]	2	2	1	5	3	2	1	1	3	0	2	1	1	24
Peng et al. (2021) [55]	1	2	1	5	3	2	2	2	3	0	2	1	1	25
Nguyen et al. (2021) [56]	2	2	1	5	3	2	2	2	3	0	2	1	1	26
Weihua Liu et al. (2021) [57]	1	2	1	5	3	2	1	2	3	0	2	1	1	24
Siqi Liu et al. (2021) [58]	1	1	1	5	3	2	1	1	3	0	1	1	0	20
Farhangi et al. (2021) [59]	2	2	1	5	3	2	1	2	3	0	2	1	0	24
Zheng et al. (2020) [60]	2	2	1	5	3	2	2	1	3	0	0	1	1	23
Xu et al. (2020) [61]	2	2	2	5	3	2	2	1	3	2	1	2	2	29
Tan et al. (2020). [62]	2	2	1	4	3	2	3	3	4	0	1	1	1	27
Ozdemir et al. (2020) [63]	1	1	1	5	3	2	1	3	3	0	2	2	1	25
Masood et al. (2020) [64]	2	2	1	5	3	2	2	2	5	0	2	1	1	28
Guo et al. (2020) [65]	2	2	2	5	3	2	2	3	5	0	2	1	1	30
Chenyang et al. (2020) [66]	1	2	1	5	3	2	2	2	4	0	1	2	1	26
Cao et al. (2020) [67]	1	2	1	5	3	2	1	3	4	0	1	1	1	25
Xie et al. (2019) [68]	2	2	1	5	3	2	3	2	4	0	1	1	1	27
Winkels et al. (2019) [69]	1	2	1	5	3	2	2	2	3	0	1	1	1	24
Nasrullah et al. (2019) [70]	2	2	1	5	3	2	3	2	3	0	0	1	1	25
Xia Huang et al. (2019) [71]	2	2	1	5	3	2	2	2	4	0	2	1	1	27
Wenkai Huang et al. (2019) [72]	2	2	1	5	3	2	3	2	3	0	0	1	1	25
Gong et al. (2019) [73]	2	2	1	5	3	2	2	2	4	0	1	1	1	26
Zhang et al. (2018) [74]	2	2	1	5	3	2	2	1	3	0	0	1	1	23
Gu et al. (2018) [75]	2	2	1	5	3	2	2	2	4	0	3	2	1	29
Setio et al. (2018) [76]	2	2	1	5	4	2	2	2	3	0	1	2	1	27

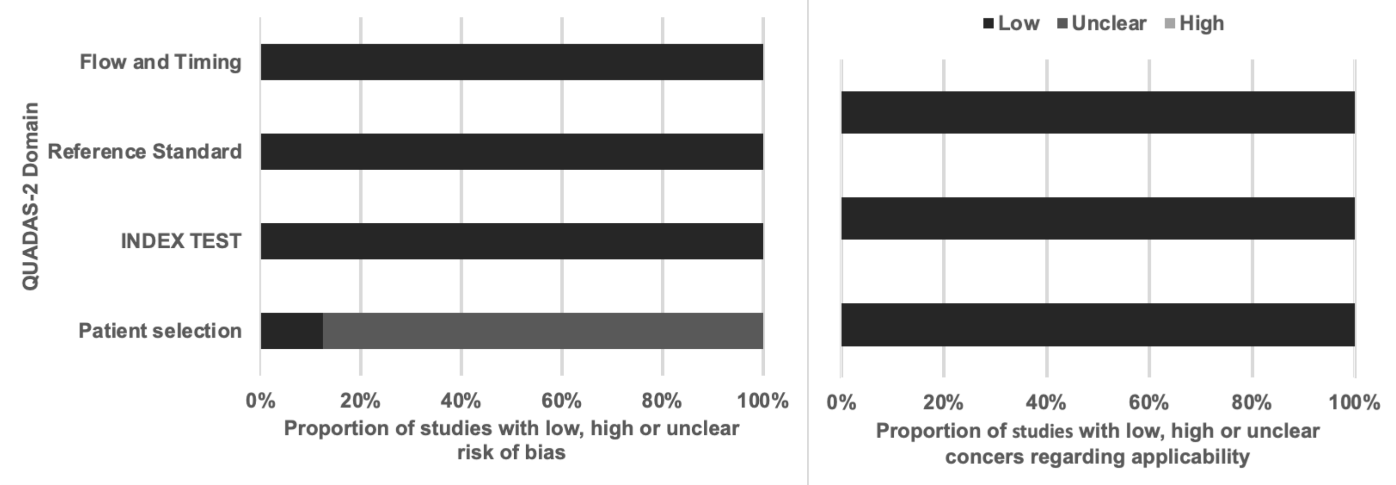

Figure S1. The results of QUADAS-2 quality assessment for included studies

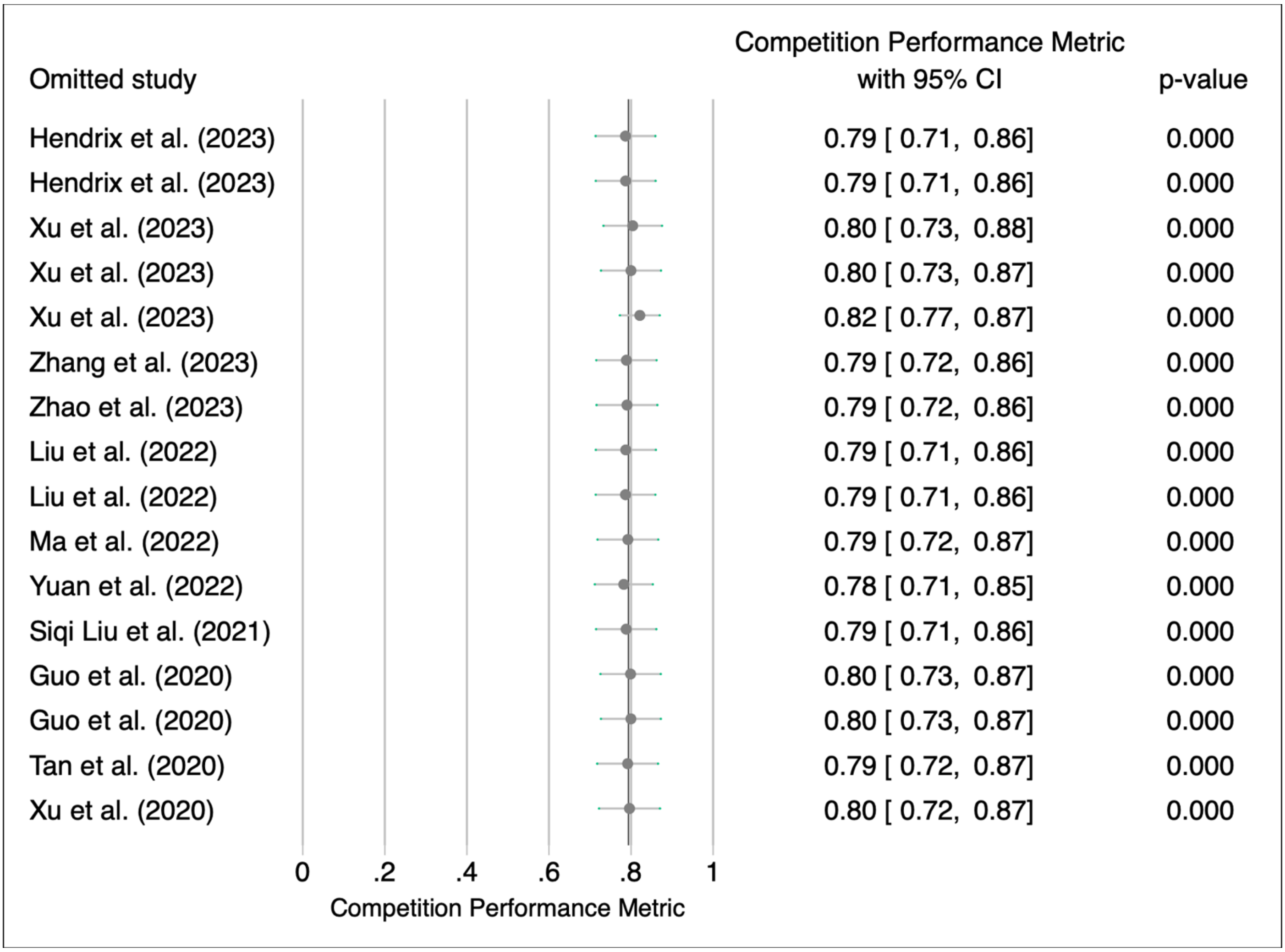

Figure S2 The results of a sensitivity analysis of deep learning algorithms' competition performance metric in independent validation dataset using the one-study removal method.

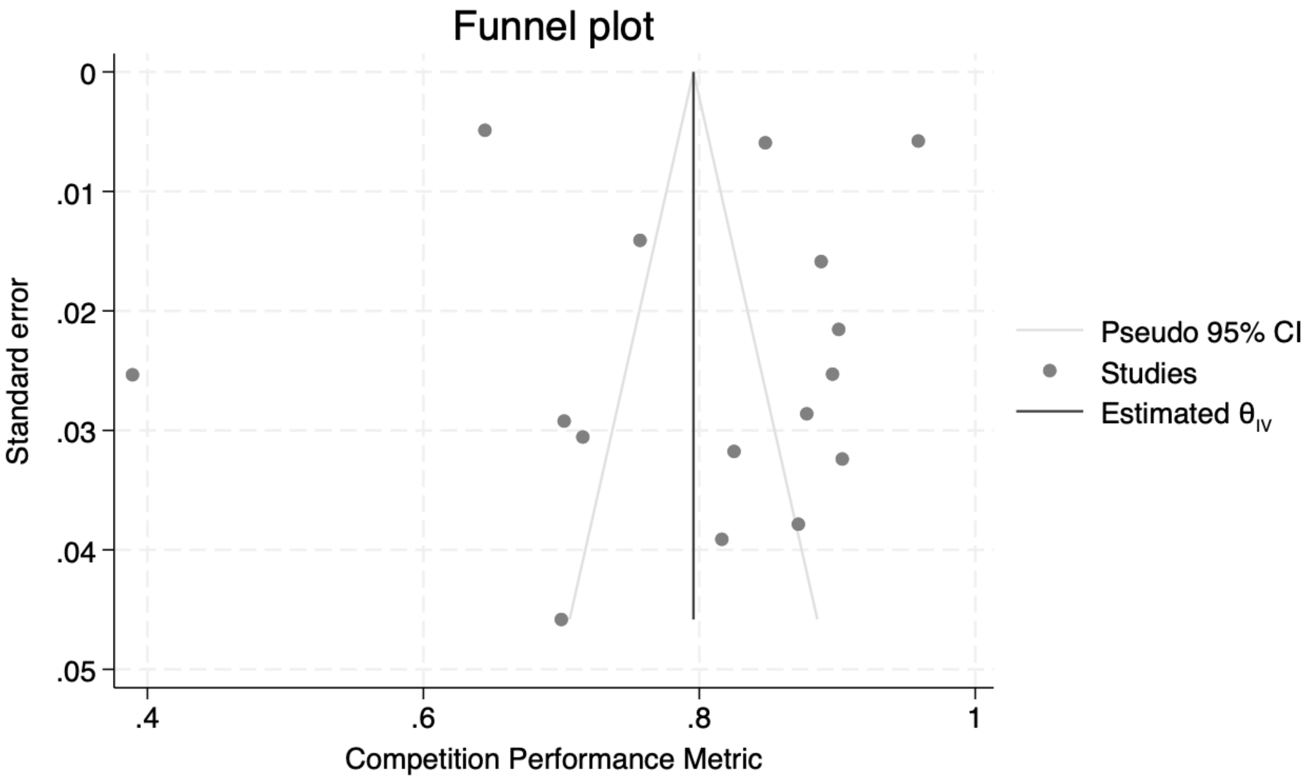

Figure S3. The funnel plot of deep learning algorithms' competition performance metric of the highest-performing result in independent validation dataset. The *p* value of the Egger's test was 0.75 indicating no publication bias.

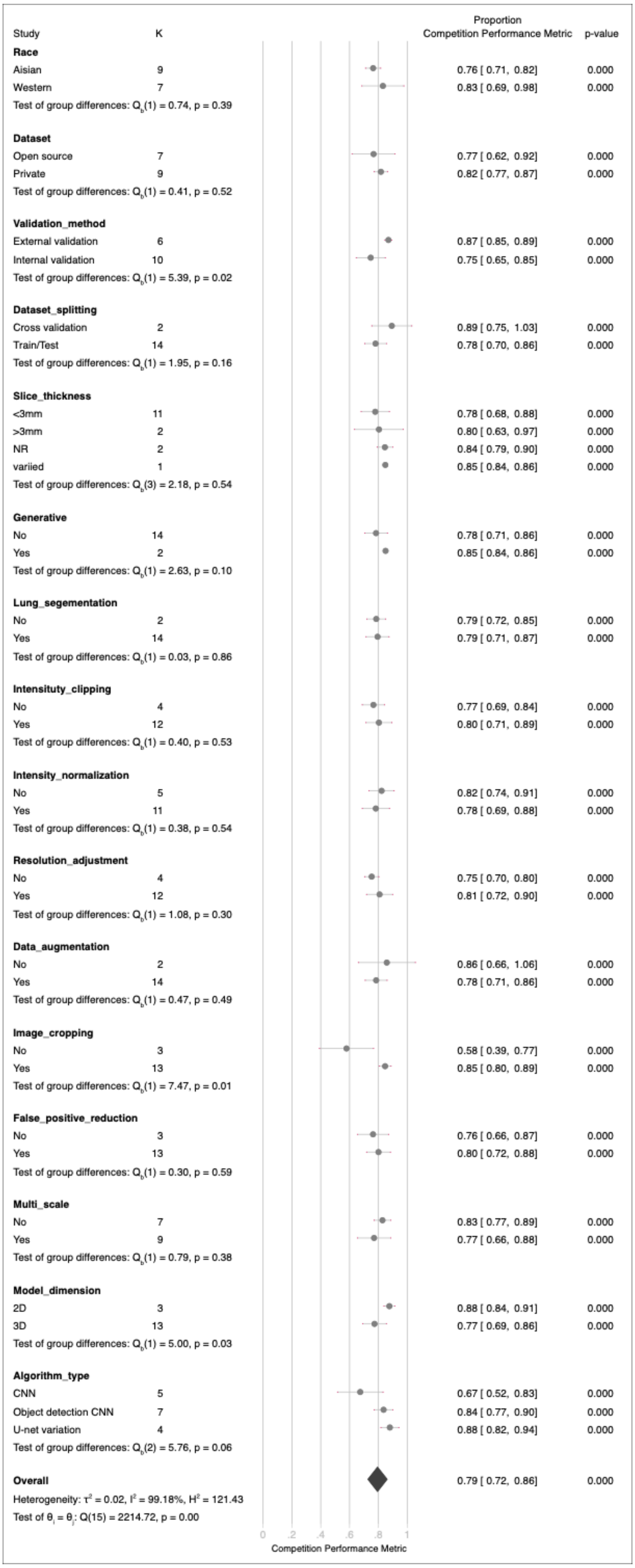

Figure S4. Forest plot of subgroup analysis competition performance metric of deep learning algorithms in independent datasets.

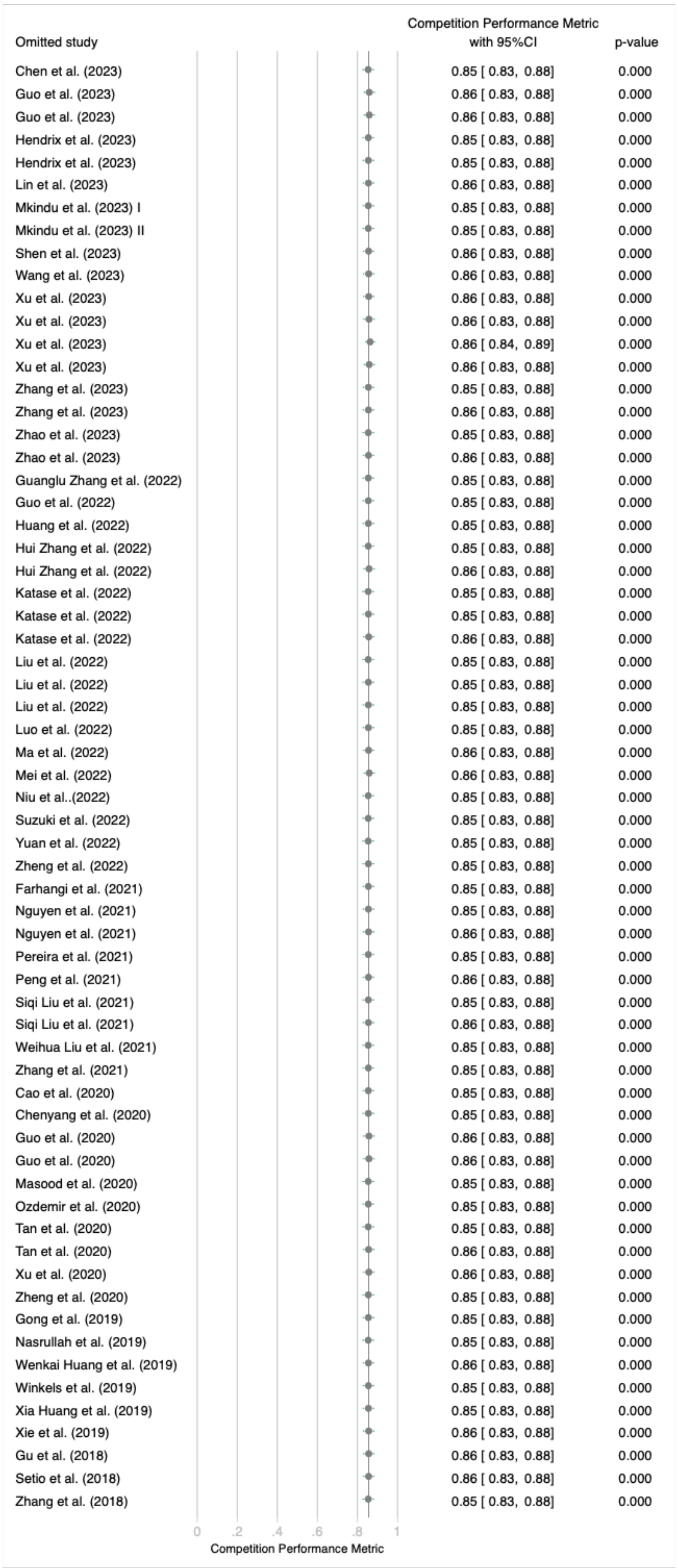

Figure S5 The results of a sensitivity analysis of deep learning algorithms' competition performance metric in all validation dataset using the one-study removal method.

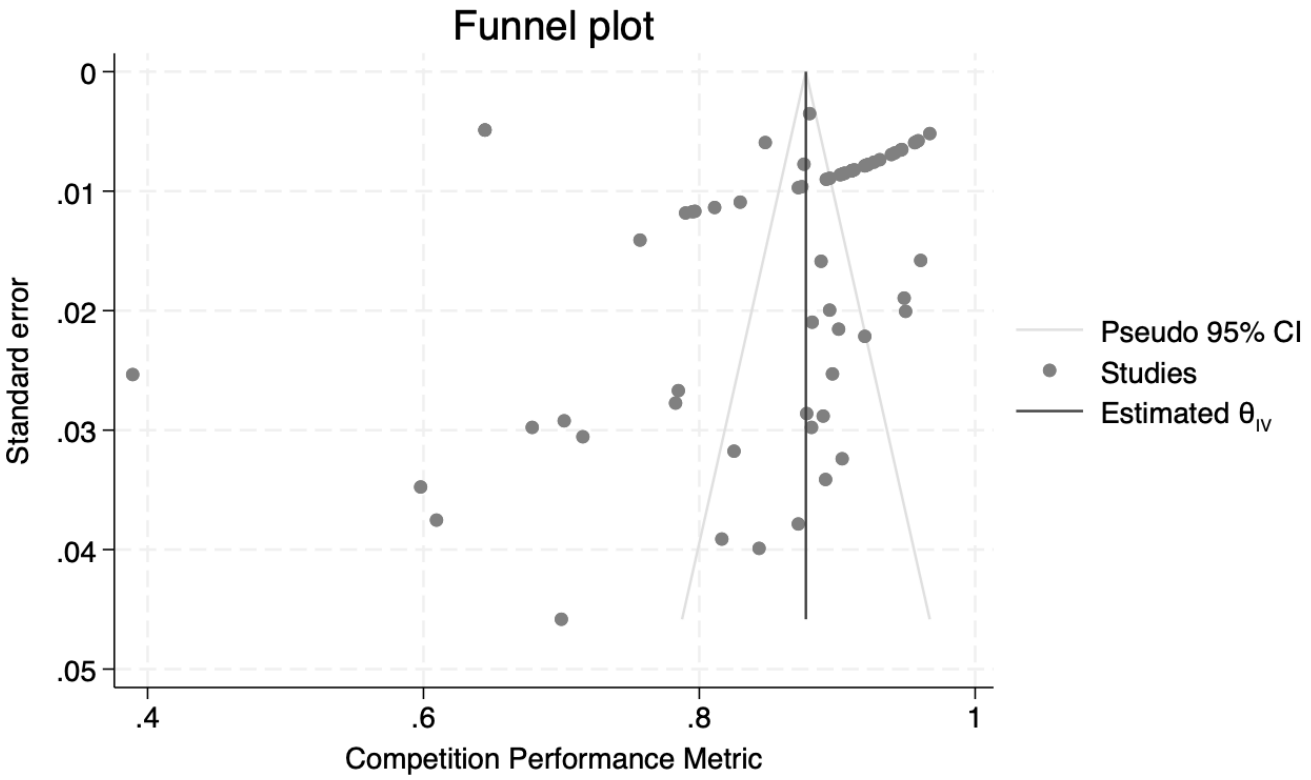

Figure S6. The funnel plot of deep learning algorithms' competition performance metric of the highest-performing result in independent validation dataset. The p-value of the Egger's test was <0.01, indicating the presence of publication bias.

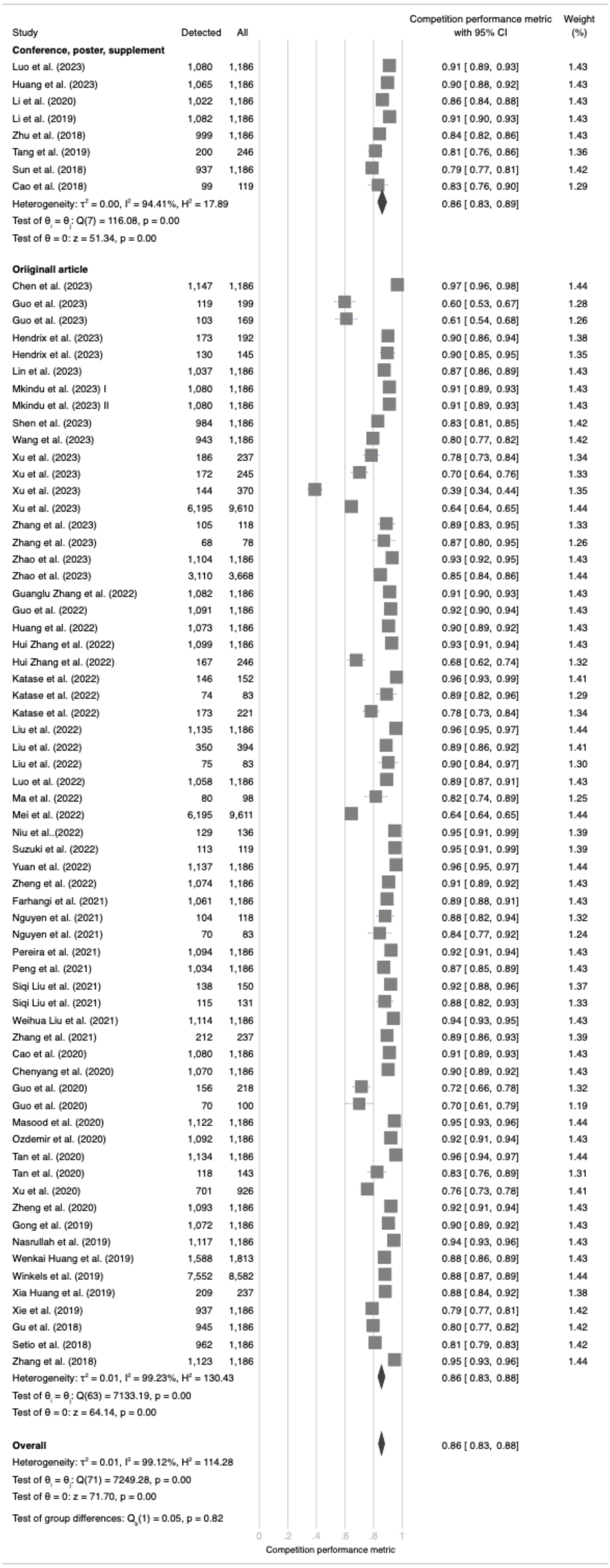

Figure S7. Forest plot of subgroup analysis competition performance metric of deep learning algorithms in independent datasets with publication status as moderator (p=0.82).
